# Use of GC–MS based metabolic fingerprinting for fast exploration of fungicide modes of action

**DOI:** 10.1186/s12866-019-1508-5

**Published:** 2019-06-24

**Authors:** Zhihong Hu, Tan Dai, Lei Li, Pengfei Liu, Xili Liu

**Affiliations:** Department of Plant Pathology, China Agricultural University, Beijing, 100193 People’s Republic of China

**Keywords:** Metabolic fingerprinting, GC–MS, *Botrytis cinerea*, Fungicide, Mode of action

## Abstract

**Background:**

The widespread occurrence of fungicide resistance in fungal plant pathogens requires the development of new compounds with different mode(s) of action (MOA) to avoid cross resistance. This will require a rapid method to identify MOAs.

**Results:**

Here, gas chromatography–mass spectrometry (GC–MS) based metabolic fingerprinting was used to elucidate the MOAs of fungicides. *Botrytis cinerea*, an important pathogen of vegetables and flowers, can be inhibited by a wide range of chemical fungicides with different MOAs. A sensitive strain of *B. cinerea* was exposed to EC_50_ concentrations of 13 fungicides with different known MOAs and one with unknown MOA. The mycelial extracts were analyzed for their “metabolic fingerprint” using GC–MS. A comparison among the GC–MS vector’ profiles of cultures treated with fungicides were performeded. A model based on hierarchical clustering was established which allowed these antifungal compounds to be distinguished and classified coinciding with their MOAs. Thus, metabolic fingerprinting represents a rapid, convenient, and information-rich method for classifying the MOAs of antifungal substances. The biomarkers of fungicide MOAs were also established by an analysis of variance and included succinate for succinate dehydrogenase inhibitors and cystathionine for methionine synthesis inhibitors. Using the metabolic model and the common perturbation of metabolites, the new fungicide SYP-14288 was identified as having the same MOA as fluazinam.

**Conclusion:**

This study provides a comprehensive database of the metabolic perturbations of *B. cinerea* induced by diverse MOA inhibitors and highlights the utility of metabolic fingerprinting for defining MOAs, which will assist in the development and optimization of new fungicides.

**Electronic supplementary material:**

The online version of this article (10.1186/s12866-019-1508-5) contains supplementary material, which is available to authorized users.

## Background

The fungal pathogen *Botrytis cinerea* causes serious losses in more than 200 crops worldwide. It can survive for relatively short periods as mycelia and/or conidia and for extended periods as sclerotia in crop debris [1]. The fungus causes grey mold disease, which can be controlled by the application of a wide range of chemical fungicides that act as seven modes of action (MOAs), including β-tubulin assembly inhibitors, respiration inhibitors, uncouplers of oxidative phosphorylation, methionine biosynthesis inhibitors, signal transduction inhibitors, sterol biosynthesis inhibitors, and multi-site inhibitors. Unfortunately, *B. cinerea* has developed high levels of resistance to most of the fungicides used for its control in the field [2–5]. Although many new fungicides that target *B. cinerea* have been developed, these may be ineffective if they have MOAs that are similar to those of fungicides to which *B. cinerea* is already resistant; i.e., there may be cross-resistance between the new fungicides and the previously used fungicides. It is, therefore, important to develop a high-throughput screening method to identify fungicide MOAs. A fast exploration of MOAs is helpful for the scientific application of new fungicides.

A series of research methods have been used to reveal fungicide MOAs. The MOA of flumorph was explored by analyzing alterations of hyphal morphology, cell wall deposition patterns, F-actin organization, and other organelles in *Phytophthora melonis*. Results showed that flumorph may be involved in the impairment of cell polar growth through directly or indirectly disrupting the organization of F-actin [6]. Deuterium-labelling was used to determine that the MOA of metalaxyl involved the inhibition of RNA polymerase I [7]. The researcher found that RNA synthesis of phenylamide-sensitive strains, measured as [3H] uridine incorporation, was inhibited by about 80% (*Phytophthora megasperma f. sp. medicaginis*) and by about 40% (*Phytophthora infestans*) by metalaxyl and oxadixyl at a concentration of 1 μg/ml. RNA synthesis of resistant strains was completely insensitive to metalaxyl and oxadixyl at concentrations as high as 200 μg/ml. Additionally, endogenous nuclear RNA polymerase activity of both *Phytophthora* sensitive isolates appeared to be more sensitive to the phenylamides than of both *Phytophthora* resistant isolates. These means of cross-resistance could be applied in the bioassay method to determine the MOA by assessing the resistance mechanism. A complex II analysis of mutants of several organisms resistant to succinate dehydrogenase inhibitors (SDHIs), such as carboxin, provided insights into the MOA of SDH-inhibitors [8, 9]. Comparison of the sequence from a carboxin-sensitive *Ustilago maydis* strain of iron-sulphur protein (Ip) subunit of succinate dehydrogenase (Sdh) with that of the Ip allele from a carboxin -resistant strain revealed a two-base difference between the sequences. This mutation led to the substitution of a leucine residue for a histidine residue within the third cysteine-rich cluster of the deduced amino-acid sequence of the Ip allele. This cluster, which is associated with the S3 iron-redox centre, is involved in the transport of electrons from succinate to ubiquinone (Q). Confirmation that this nucleotide change led to enhanced resistance to carboxin was obtained following mutagenesis of the sensitive Ip allele to the resistant form and expression of the mutated allele in *U. maydis* [8]. A patent proposed the use of affinity chromatography to determine the MOA of oxathiapiprolin [10]. The authors found that the oxathiapiprolin specifically binds to *Oomycete* oxysterol binding polypeptide in the total protein mixture obtained from *Oomycete*. The MOA of quinone outside Inhibitors (QoI) was explored using protein crystallization combined with molecular docking. The existence of more than 40 different fungicide MOAs (FRAC, 2019) makes screening by the methods above time-consuming and costly. Thus, fast, robust, and high-throughput screening techniques are required.

In recent years, the application of omics approaches has greatly accelerated the progress of MOA identification for new fungicides. For example, the MOA of phenamacril against *Fusarium graminearum* was studied by the high-throughput sequencing of the fungal genome and transcriptome [11], and proteomics was applied to study the MOA of pyrimorph [12]. To determine the mechanism of resistance to the fungicide phenamacril in *F. graminearum*, the researchers sequenced and annotated the genome of the resistant strain YP-1. They sequenced 22 functional annotated genes of another *F. graminearum* sensitive strain (strain 2021) and corresponding resistant strains. The only mutation common to all of the resistant mutants occurred in the gene encoding myosin-5 (point mutations at codon 216, 217, 418, 420, or 786). Further, they found that transformed mutants with a copy of the resistant myosin-5 locus fragment exhibited resistance to phenamacril, and the transformed mutant with a copy of the sensitive fragment exhibited sensitivity to phenamacril [11]. The proteomic response of *Phytophthora capsici* to pyrimorph was investigated using the iTRAQ technology to determine the target site of the fungicide and potential biomarker candidates of drug efficacy. Many of the proteins with altered expression were associated with glucose and energy metabolism. Biochemical analysis using D-[U-14C] glucose verified the proteomics data, suggesting that the major MOA of pyrimorph on *P. capsici* is the inhibition of cell wall biosynthesis [12]. Metabolomics involves the comprehensive qualitative and quantitative profiling of a large number of metabolites of a biological system. The major advantage is the simultaneous monitoring of metabolic networks, which enables their changes to be associated with biotic and/or abiotic causal agents and enables the detection of corresponding biomarkers. The metabolic footprinting of *Saccharomyces cerevisiae* provided sufficient information to discern the MOAs of antifungal substances. Respiratory inhibitors were discriminated from other kinds of inhibitors, while relationships between metabolites and MOAs were not described in detail [13]. The use of metabolomics for the study of fungicides MOAs is still in its infancy [14]. Thus, metabolomics is a powerful tool to identify the MOAs of antifungal and antibiotic compounds by fingerprinting model discrimination, biomarker identification, and metabolic pathway excavation.

In this paper, the effects of different fungicide MOAs on the metabolome of *B. cinerea* were studied. A metabolic fingerprint model was established, the use of biomarkers was investigated, and the MOA of a novel fungicide, SYP-14288, was predicted.

## Results

### Sensitivity of *B. cinerea* to fungicides

The EC_50_ values of the 14 fungicides tested against SP2–6 are shown in Table 1. They ranged from 0.004 μg/mL to 5.41 μg/mL which indicated that SP2–6 has a good sensitivity to the fungicides. Among the fungicides tested, fluazinam and SYP-14288 showed the greatest inhibitory activities, with EC_50_ values lower than 0.01 μg/mL.

**Table 1:** EC_50_ values of different mode of action fungicides to *B. cinerea* SP2–6

Mode of action group	Fungicides	CAS	EC_50_ (μg/mL)	Concentrations used (μg/mL)
β-tubulins assembly	Carbendazim	10,605–21-7	0.04	0, 0.005, 0.013, 0.05, 0.1, 0.2
	Thiophanatemethyl	23,564–05-8	0.36	0, 0.2,0.3, 0.4, 0.6, 0.9
Respiration	Boscalid	188,425–85-6	0.08	0, 0.02, 0.05, 0.1, 0.5, 1
	Kresoxim-methyl	143,390–89-0	0.13	0, 0.02, 0.04, 0.08, 0.78, 1.56
	Fluazinam	79,622–59-6	0.01	0, 0.001, 0.005, 0.05, 0.1, 0.2
Mitochondrial function	Cyprodinil	121,552–61-2	0.47	0, 0.15, 0.3, 1, 3, 15
	Pyrimethanil	53,112–28-0	0.68	0, 0.01, 0.5, 1, 2, 5
Signal transduction	Fludioxonil	131,341–86-1	0.02	0, 0.005, 0.01, 0.02, 0.04, 0.05
	Procymidone	32,809–16-8	0.39	0, 0.23, 0.45, 0.6, 0.8, 0.9
Membrane sterol biosynthesis	Imazalil	35,554–44-0	4.64	0, 1, 2.5, 5, 10, 15
	Fenhexamid	126,833–17-8	0.41	0, 0.1, 0.25, 0.5, 1, 5
Multi site action	Chlorothalonil	1897-45-6	5.41	0, 0.1, 0.5, 1, 10, 50
	Thiram	137–26-8	1.59	0, 0.39, 0.78, 1.56, 3.13, 6.25
Unknown	SYP-14288		0.004	0, 0.001, 0.003, 0.005, 0.01, 0.05

Note: The EC_50_ values of fungicides against *B. cinerea* SP2–6 were measured based on the mycelial growth rate method on PDA plates. Each combination of isolate and fungicide concentration was represented by 6 replicate plates, and the experiments were performed 3 times

### Metabolome of *B. cinerea*

**Fig. 1:**
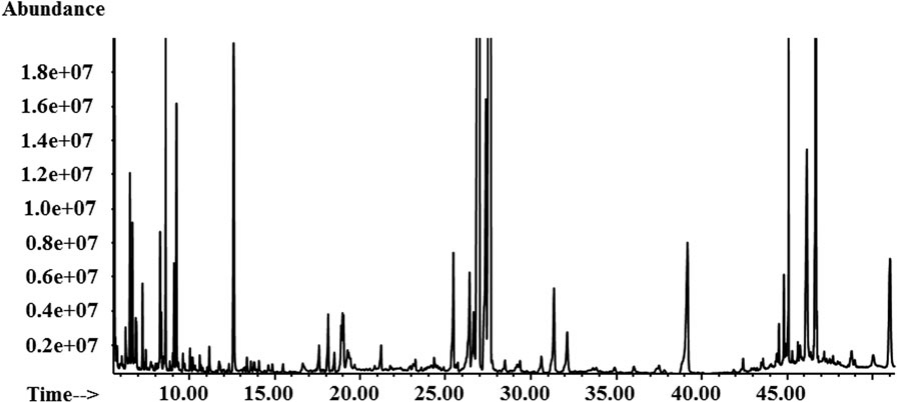
Total ion current chromatogram from a typical analysis of the metabolome of *B. cinerea* SP2–6. Abundance means the amount of ion. Time means retention time / min

The *B. cinerea* mycelia was grown on PDA plates supplemented individually with 14 fungicides (EC_50_) and DMSO (0.1% v/v, control) for 3 days. After incubation, the metabolites of the homogenized mycelia were extracted, dried and derivatized by methoximation and trimethylsilylation prior to GC–MS analysis. A typical total ion current chromatogram is shown in Fig. 1. The detected *B. cinerea* metabolome consisted of 245 metabolites based on NIST 2005, including mainly amino acids, organic acids, alcohols, sugars, alkanes, and organic esters. The relative standard deviation (RSD) of the internal standard substance salicin was 3.49%, which indicated the acceptable stability of the instrument. The RSDs of the metabolites in the 6 technical replicates ranged from 3.53 to 245%, which was in accordance with a previous report [15].

### HCA of the metabolic fingerprint of *B. cinerea* treated with fungicides having different MOAs

**Fig. 2:**
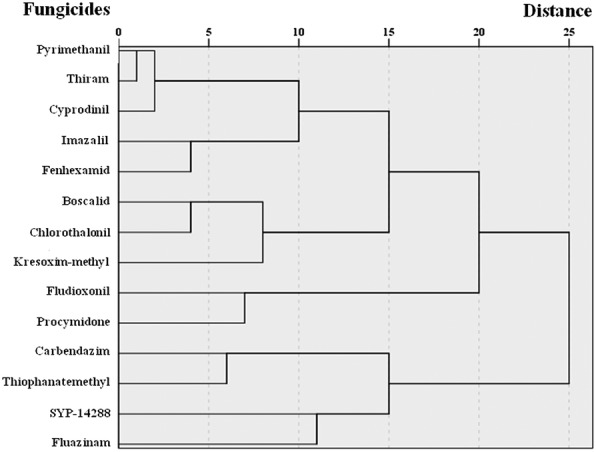
Clustering analysis results of the studied fungicides by metabolic fingerprinting. 179 metabolites of 6 replicates went into the clustering analysis. Based on metabolites and their relative contents of *B. cinerea* SP2–6, most of the 14 fungicides were discriminated as 6 groups which coincided with their modes of actions in FRAC

The HCA was dominated by six distinct groups that were very similar to groups based on MOAs in FRAC (Fig. 2, Additional file 1). One group contained the methionine biosynthesis fungicides pyrimethanil and cyprodinil, multi site action inhibitor thiram. The second group contained the fungicides imazalil and fenhexamid that interfered with sterol biosynthesis. The third group contained the respiration inhibitors fungicides boscalid and kresoxim-methyl, multi site action inhibitor clorothalonil. The fourth group contained the fungicides fludioxonil and procymidone that interfered with signal transduction. The fifth group contained the fungicides carbendazim and thiophanatemethyl that act during β-tubulins assembly. The last group contained the uncoupler of oxidative phosphorylation fluazinam and the MOA unknown fungicide SYP-14288.

### The *B. cinerea* metabolome fluctuates after exposure to fungicides having different MOAs

The concentration and composition of metabolites were compared between the fungicide treatment and control groups using the one-way ANOVA followed by a Tukey’s test at a significance level of *p* < 0.05. In response to treatment with different chemicals, the metabolome of *B. cinerea* fluctuated, the number of detected metabolites ranging from 11 to 83. Table 2 shows the amount of metabolites specifically and commonly up-regulated and down-regulated after treatment with different fungicides within the same MOA group (Additional file 2).

**Table 2:** Changes in SP2–6 metabolites after exposure to different fungicides

Mode of action group	Fungicides	Number of metabolites changed (up, down)	
		Specifically changed	Commonly changed
β-tubulins assembly	Carbendazim	50 (48, 2)	8 (7, 1)
	Thiophanatemethyl	24 (16, 8)	
Respiration	Boscalid	27 (15, 12)	11 (6, 5)
	Kresoxim-methyl	26 (22, 4)	
	Fluazinam	66 (62, 4)	
Mitochondrial function	Cyprodinil	4 (1, 3)	17 (1, 16)
	Pyrimethanil	8 (1, 7)	
Signal transduction	Procymidone	22 (21, 1)	25 (15, 10)
	Fludioxonil	55 (44, 11)	
Membrane sterol biosynthesis	Fenhexamid	14 (10, 4)	7 (2, 5)
	Imazalil	4 (2, 2)	
Multi site action	Chlorothalonil	83 (65,18)	n.a.
	Thiram	37 (15, 22)	n.a.
Unknown	SYP-14288	20 (15,5)	n.a.

Note: The concentration and composition of metabolites were compared between the fungicide treatment and control groups using the one-way ANOVA followed by a Tukey’s test at a significance level of *p* < 0.05. Metabolites changed column showed the number of metabolites specifically (fungicide specific unique) and commonly regulated within the same mode of action group after treated by different fungicides. “n.a.” means not analysed

The results that fungicides of the same MOA share a large number of commonly changed metabolites indicated that they disturbed similar metabolic pathways. In this research, the two fungicides affecting mitochondrial processes [16] and amino acid biosynthesis, cyprodinil and pyrimethanil, induced down-regulation of 16 identical metabolites and up-regulation of 1 identical metabolites, and the two fungicides affecting signal transduction related to osmoregulation, procymidone and fludioxonil, induced up-regulation of 15 and down-regulation of 10 identical metabolites.

At the same time, the existence of the specifically changed metabolites of *B. cinerea* by each fungicide may be related to their belonging to different MOA subgroup and the difference in chemical structures. For instance, boscalid, kresoxim-methyl and fluazinam, belonging to three subgroups as inhibition of complex II, complex III cytochrome bc1(ubiquinol oxidase) at Qo site and uncouplers of oxidative phosphorylation, induced 27, 26 and 66 specifically changed metabolites in *B. cinerea*. For carbendazim and thiophanatemethyl, although they are both from the same subgroup as β-tubulin inhibitors, the chemical group of the latter has been reported to act with toxicity only after metabolism in organism [17]. This may be a reason to explain the specifically changed metabolites difference as 50 and 24 induced by the two fungicides.

### Biomarker analysis of succinate dehydrogenase-inhibitors and methionine biosynthesis-inhibitor

**Fig. 3:**
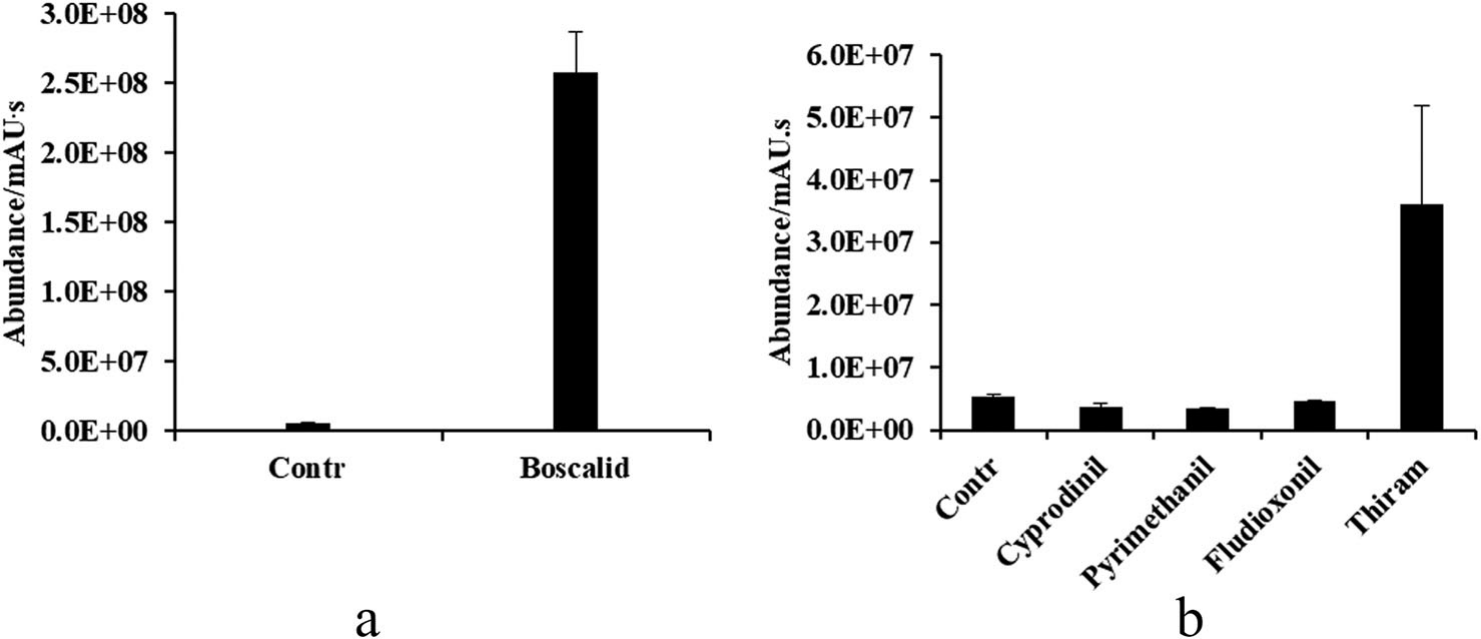
Amount of succinate in *B. cinerea* mycelium after treatment with succinate dehydrogenase-inhibitors (**a**) and other inhibitors (**b**). Contr is the control value. Each value is the mean after 6 replicates ± standard deviation. Abundance/mAU.s means the unit of peak area

In this study, succinate content specifically increased both in boscalid and thiram treated mycelium. In contrast, succinate levels were downregulated after treatment with cyprodinil, pyrimethanil and fludioxonil, or they remained unaffected in the presence of other fungicides. More than 40 times of upregulation was detected in the mycelia of SP2–6 treated by boscalid (Fig. 3a, b).

**Fig. 4:**
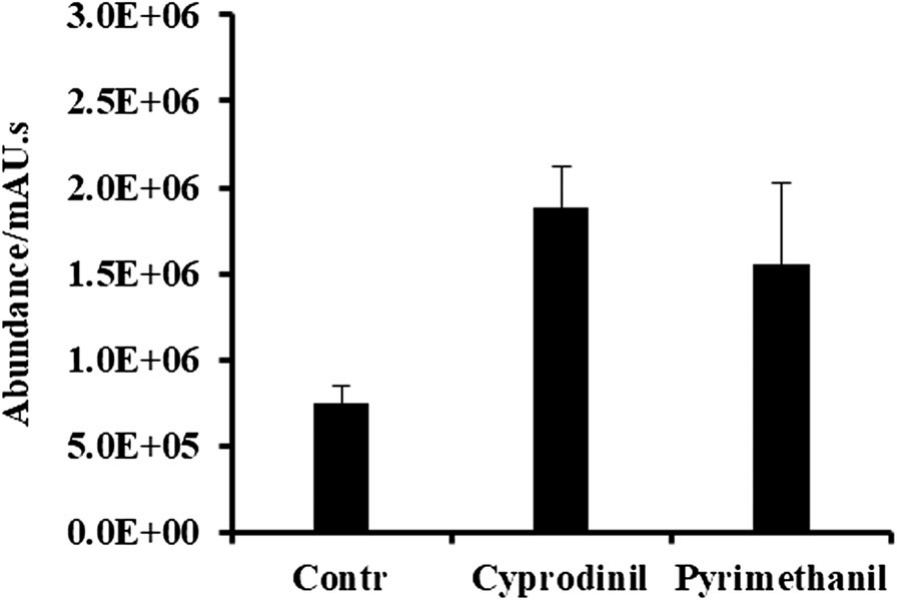
Amount of cystathionine in *B. cinerea* mycelium after treatment with methionine biosynthesis-inhibitor. Contr is the control value. Each value is the mean after 6 replicates ± standard deviation. Abundance/mAU.s means the unit of peak area

The one-way ANOVA (followed by a Tukey’s test at a significance level of *p* < 0.05) of metabolites in SP2–6 treated with or without methionine biosynthesis inhibitors showed that the total amount of cystathionine increased significantly. It increased 1.1 and 1.5 times in the mycelia of SP2–6 after pyrimethanil and cyprodinil treatments, respectively (Fig. 4). Treatments with other fungicides did not lead to significant changes in cystathionine contents.

### The preliminary prediction MOA of SYP-14288

In addition, the ANOVA analysis showed that SYP-14288 induced metabolite changed were similar to those caused by fluazinam. Among the 20 metabolites with significant differences after treatment with SYP-14288, 18 were common to fluazinam treatments. The contents of 15 metabolites were increased, while the other 3 were reduced.

## Discussion

### Sensitivity of *B. cinerea* to fungicides

A sensitive strain is necessary to explore the MOAs of fungicides. The compounds commonly combine with their target proteins effectively and inhibit the enzymes’ activities. Here, we selected the strain SP2–6, which is susceptible to the MOAs of typical fungicides used for the control of grey mold in the field, such as carbendazim, and pyrimethanil. We inferred the level of susceptibility according to the MIC of fungicides. In addition, the strain is presumed to be sensitive to fungicides of the same MOA when sensitive to a certain fungicide.

### Discrimination of MOAs by HCA

The HCA of the metabolic profile of *B. cinerea* after fungicides treatments classified most of the test fungicides into groups that showed good agreement with the six types of MOAs. Thus, the metabolites of *B. cinerea* may reflect the characteristics of fungicides with similar MOAs.

The relationships between multi-site fungicides and respiration inhibitors or methionine biosynthesis inhibitors were close. Multi-site fungicides caused a wider range of metabolic differences and, to some extents they had similar metabolic differences compared with respiration inhibitors and methionine biosynthesis inhibitors.

In this study, the metabolic fingerprints of the SP2–6 mycelia grown on PDA plates supplemented with fungicides having similar targets clustered together. The clustering analysis of the metabolic fingerprinting reflected the MOAs of the tested fungicides. We can assume that HCA clustering can be performed using the above system when speculating on the MOA of fungicides. Fungicides with unknown MOA will be aggregated with ones of similar MOA to provide an effective reference for our prediction. Thus, the metabolic fingerprints of fungicides have the potential to be used to group fungicides based on their MOAs.

The results are consistent with a report [13] on the separation of non-respiratory inhibitors from respiratory inhibitors, and complex II and III respiratory inhibitors from sterol biosynthesis inhibitors. However, β-tubulin assembly inhibitors and signal transduction inhibitors did not follow the rule. The uncoupler formed a separate class in this study, which is different from the literature in which fluazinam and sterol biosynthesis inhibitors form a group. However, fluazinam is distant from the other respiratory inhibitors and this is related to its MOA being different from those of the other inhibitors.

### Biomarkers of fungicide MOAs

Metabolites that vary significantly in the fungicide treatment group may be directly or indirectly related to the mechanism of action, and some specific metabolites of which can be identified as potential biomarkers of certain fungicide MOAs.

### Succinate dehydrogenase inhibitors

In this study, the content of succinate increased more than 40 times in the mycelia of SP2–6 after the boscalid treatment. This accumulation is in accordance with the mechanism. Boscalid is a succinate dehydrogenase inhibitor that targets the succinate dehydrogenase complex in the respiratory chain [18]. This enzyme complex couples the oxidation of succinate to fumarate in the mitochondrial matrix (or in the cytoplasmic membrane of pathogens) with the reduction of ubiquinone to ubiquinol in the membrane during aerobic respiration [19]. The fungicide interferes with the succinate dehydrogenase complex to block the oxidation of succinate to fumarate and affects the normal life activities of pathogens. It is speculated that the succinate content increased in the mycelia of SP2–6 after the multi-site action inhibitors thiram treatment related to the mechanism of it acting on the electron transport chain. However, the succinate content decreased significantly after cyprodinil, pyrimethanil and fludioxonil treatment. Thus, succinate can be a good biomarker of succinate dehydrogenase inhibitors.

### Methionine biosynthesis inhibitors

In this study, the total amount of cystathionine increased significantly in metabolites of SP2–6 treated with pyrimethanil and cyprodinil. This was consistent with previous research that pyrimethanil and cyprodinil may be disturbing the action of cystathionine β-synthase on synthesis of methionine from cystathionine [4]. However, another research has showed that anilinopyrimidine primarily target the mitochondria. Thus, the role of cystathionine as a biomarker identified by metabolomics remains to be further verified.

### Predicting SYP-14288’s MOA

SYP-14288 and fluazinam treatment induced similar regulation in metabolic level, SYP-14288, belonging to the diarylamines, has a structure similar to that of fluazinam [20]. It is suggested that the MOA of SYP-14288 is similar to that of fluazinam, which act as an uncoupler of oxidative phosphorylation [21].

SYP-14288 and fluazinam were clustered into one group (Fig. 2), indicating that they share the same MOA. We can safely predict that SYP-14288 is an uncoupler of oxidative phosphorylation since the MOA recognition through clustering has been reported several times in medical research. Metabolic profiles of *Staphylococcus aureus* after treatments with antibiotics having known MOAs have been compared with those obtained from treatments with the antibiotic compounds being tested. Using this platform, Yu et al. [22] performed a principal components analysis (PCA) and revealed that the antibacterial MOAs of rhizome extracts of the plant *Tinospora capillipes* Gagnep. resembled those of rifampicin and norfloxacin, which act on nucleic acids. Similarly, Yi et al. [23] performed a PCA and found that the MOA of berberine may be similar to that of rifampicin or norfloxacin. Also using *S. aureus* as the model organism, Liu et al. [24] performed GC–MS metabolomics in combination with HCA and concluded that the MOAs of the four synthesized antibiotics under study resembled that of clindamycin, which inhibits protein synthesis by reversibly binding to the 50 S subunit of ribosomes. Thus, the MOAs of new agents can be explored based on metabolome and cluster analyses.

We have evidence which makes it plausible that at least one MOA of SYP-14288 may be an uncoupler of oxidative phosphorylation. However, we still need to further verify and analyze the metabolic pathways affected by SYP-14288 and related enzymes.

## Conclusions

Here, we described a comprehensively and quickly exploring approach for determining the MOAs of unknown antifungals using the microbial metabolic fingerprinting models for fungicides with known MOAs. First, we tested this hypothesis in a *B. cinerea* model, using mycelia as the target and 13 fungicides as exogenous metabolic challenges. Second, we analyzed fungicides having the same MOAs by HCA or by comparing normal mycelia to mycelia that were exposed to a sub-lethal dose of SYP-14288 to identify biomarkers. Theoretically, this metabolomics methodology could be extended (1) to predict tentative MOAs for new fungicides, and (2) to identify the disturbed metabolic pathways.

The HCA was performed based on the *B. cinerea* metabolome and these 13 fungicides correctly clustered in to 6 different MOA groups. The biomarkers for succinate dehydrogenase and methionine synthesis inhibitors were obtained using a one-way ANOVA between treatments and control metabolites. Not only did SYP-14288 clustered near fluazinam, but also a large number of similar metabolome changes occurred in both of them, indicating that these two fungicides may have a similar MOA.

The metabolome fluctuations mirrored the changes downstream of the pathogen under disturbance, but the targets of the fungicides need to be determined using molecular biology and biochemistry. Compared with the control group, the metabolic fingerprint of SP2–6 changed after being exposed to fungicides. The metabolic fingerprints differed among different fungicide treatments. These altered metabolites were correlated with the fungicides’ MOAs and have the potential to be used as MOA biomarkers. In addition, fungicides having similar MOAs had short clustering distances and the changes in the metabolic fingerprint of SP2–6 can reflect the MOAs. Thus, metabolic fingerprinting can be a promising approach to quickly gain insight in to the potential MOAs of fungicides, which then can direct the follow up validation work using molecular genetic tools.

## Methods

### Chemicals and materials

The 14 fungicides (with known and unknown MOAs) included the β-tubulin assembly inhibitors carbendazim (Sichuan Guoguang Agrochemicals Co., Ltd) and thiophanatemethyl (Hainan Li Zhi Biological Co., Ltd.), the respiration inhibitors complex II succinate dehydrogenase inhibitor boscalid (Beijing Tak Lida Agricultural Science and Technology Co., Ltd), quinone outside inhibitor kresoxim-methyl (Shandong Jingbo agricultural companies), the uncoupler of oxidative phosphorylation fluazinam (Jiangsu Youshi Chemical Co., Ltd), the methionine biosynthesis inhibitors cyprodinil (Beijing Mingde Rieter Agriculture Branch Co., Ltd) and pyrimethanil (Tianjin Shi Pu Le Pesticide Technology Development Co., Ltd), the signal transduction inhibitors fludioxonil (Shandong Weifang Runfeng Chemical Co., Ltd), and procymidone (Lier chemical co., Ltd), the membrane sterol biosynthesis inhibitors imazalil (Liuzhou Huinong Chemical Co., Ltd) and fenhexamid (Ai Li Sida Biological Chemicals Co., Ltd), and the multi-site action inhibitors chlorothalonil (Jiangyin Suli Chemical Co., Ltd) and thiram (Yingkou Leike Pesticide Co., Ltd.) (Table 1). The fungicides were from commercial sources with a minimum purity of 95%. SYP-14288, a new fungicide with an unknown MOA, which was created by imitating fluazinam, came from the Shenyang Chemical Industry Co. Ltd. The fungicides were dissolved in dimethyl sulfoxide (DMSO; Xilong Chemical Co., Ltd.) to produce 10^4^ μg/mL stock solutions. From these stocks, other additional stock concentrations were prepared by serial dilution. Stocks were aliquoted into 2 mL sterile microcentrifuge tubes and stored at − 4 °C in the dark.

Methanol (chromatographically pure) was purchased from the Sinopharm Chemical Reagent Co., Ltd. Ultrapure water was obtained from a milli-Q system (Millipore). *N*, *O*-Bis (trimethylsilyl) trifluoroacetamide (v/v; contains 1% trimethylchlorosilane (TMCS), 99%), pyridine (99.8%), methoxyamine hydrochloride (98%), internal standard salicin and 40 other metabolite standards with active ingredients greater than 99% were purchased from Sigma-Aldrich.

### Sensitive strain selection and EC_50_ measurements

*B. cinerea* strain SP2–6, which is sensitive to 6 fungicides MOAs, was selected from 115 isolates taken from tomato plants suffering grey mold in 8 Chinese provinces from 2006 to 2015. The isolates were identified as *B. cinerea* based on morphology and the DNA sequence data of three nuclear protein-coding genes glyceraldehyde-3-phosphate dehydrogenase (*G3PDH*), Heat-shock Protein 60 (*HSP60*), and DNA-dependent RNA polymerase subunit II (*RPB2*) [25]. First, the sensitive *B. cinerea* strain were selected by observing the failure to grow on PDA plates independently supplemented with the minimum inhibitory concentration (MIC) of carbendazim, thiophanatemethyl, boscalid, cyprodinil, procymidone, fludioxonil, and fenhexamid [26–28]. Then, EC_50_ values of kresoxim-methyl, imazalil, and pyrimethanil were tested and then sensitivity baselines [29] were used to screen for sensitive strains. There are limited resistance reports for fluazinam and the multi-site fungicides chlorothalonil and thiram, therefore, no sensitivity determinations were conducted.

The EC_50_ values of fungicides against strain SP2–6 were measured based on the mycelial growth rate method on PDA plates [30]. The stock solutions were added to molten PDA media when they cooled to approximately 50 °C. For kresoxim-methyl, sensitivity tests were conducted in the presence of 100 μg/mL salicylhydroxamic acid, a specific inhibitor of alternative oxidases.

The strain SP2–6 was grown on PDA plates for 4 days at 20 °C in the dark before the mycelial plugs were transformed from the colony margin to the center of PDA plates independently supplemented with a serial dilution of a fungicide. To assist their dissolving, DMSO was used at a final concentration of 0.1% in the medium. Each combination of isolate and fungicide concentration was represented by 6 replicate plates, and the experiments were performed 3 times. After 4 days of cultivation, the radial growth was recorded to calculate the percentage of growth inhibition (relative to the growth on plates without fungicides). EC_50_ values were calculated by the regression of the probit of the percentage of inhibition of radial growth against the logarithmic values of fungicide concentrations.

### Mycelial sample collection

Mycelial samples of the strain SP2–6 were collected after growing on plates containing the 14 fungicides or DMSO (control). The experimental procedure was as follows: The strain SP2–6 was grown on PDA plates for 3 days at 20 °C in the dark. A mycelial plug was taken from the colony margin with a cork borer (5 mm) and placed mycelial-side down in the center of a PDA plate amended with the EC_50_ of a fungicide and covered by cellophane (diameter of 4.2 cm). For control groups, the mycelia were cultured on PDA plates with a 0.1% final concentration of DMSO. Each combination of isolate and fungicide concentration was represented by 6 replicate plates, and the experiments were performed three times. After 3 days at 20 °C, all of the mycelia except the plug on the cellophane were harvested with scalpels into 2 mL centrifuge tubes, frozen with liquid nitrogen, and stored at − 80 °C. At the time of sampling, the mycelia cultivated on plates supplemented with fungicides had almost extended to the margin of the cellophane, while the mycelia cultivated on plates not supplemented with a fungicide (DMSO only) had already grown over the cellophane margin.

### Metabolite extraction and derivatization

#### Extraction

The free polar metabolites were extracted in the supernatants of water–methanol solutions of the homogenized biological samples according to the literatures [31, 32] with some change as follows.

The mycelial samples stored at − 80 °C were homogenized by ball milling at 30 times/s for 2 min after soaked in liquid nitrogen. The sample aliquots were mixed with 1.8 mL of solvent mixture (methanol/water, 80/20, v/v) containing internal standard salicin at 10 μg/mL, stored at least for 30 min on ice), vortexed vigorously for 1 min and then placed in an ultrasound water bath held at 25 °C (velocity of ultrasound in water (1500 m/s)/frequency (40 kHz) = 3.75 cm) for 15 min. The samples were centrifuged at 14000 g for 15 min. Then, 0.6 mL of supernatant solution was dried in a vacuum centrifuge at 45 °C. The acquired dried metabolite mixture was then used for further analyses.

#### Derivatization

For derivatization, 100 μL of methoxylamine hydrochloride in pyridine (20 mg/mL) was added to the dried extractions prior to incubation at 30 °C for 2 h. Then, 100 μL of N, O-bis (trimethylsilyl) trifluoroacetamide containing 1% trimethylchlorosilane (v/v) was added, and the mixtures were incubated at 37 °C for 6 h. After centrifugation at 14000 g for 15 min, 160 μL of supernatant was transferred to vials for detection. All of the solutions were tested within 48 h.

### GC–MS

The GC–MS analysis was performed on a QP 2010 GC (Agilent Technologies 6890 N)–MS (Agilent Technologies 5973 i) system using a HP-5MS (30 m × 0.25 mm × 0.25 μm) capillary column. The injector temperature was set to 280 °C with a split ratio of 5. Helium was employed as carrier gas at 1 mL/min. The column temperature was initially kept at 65 °C for 2 min. It was then increased to 185 °C by 5 °C min^− 1^, increased to 200 °C by 1 °C min^− 1^, and increased to 280 °C by 15 °C min^− 1^, where it was held for 25 min. The MS scan parameters were set as follows: a mass scan range of m/z 20–650, a scan interval of 0.2 s, and a detector voltage of 0.9 kV. The ion source temperature was 230 °C, and the interface temperature was 280 °C. The solvent cut time was 5.5 min. The injection volume was 1 μL, constant current mode was used, and the MS electron ionization source was 70 eV. The GC–MS data (Enhanced Data Analysis MSD ChemStation Build 75, 26-Aug-2003) were analyzed and qualitative and quantitative data on retention times, names, match qualities were exported by the software. The integrator was that of ChemStation. In the MS Signal Integration Parameters, for Edit Integration Events, the Initial Area Reject was 1, Initial Peak Width was 0.020, Shoulder Detection was OFF, and Initial Threshold was 18.0. In the total ion current chromatogram of a typical sample, one characteristic ion and two reference ions were selected as the qualitative ions to edit the information for all of the metabolites. The names and structures of the metabolites were retrieved from the NIST 2005 database and standards. The structures of the metabolites were based on the reaction rules of metabolites with derivatization reagents [33].

### Statistical analyses

The instrument stability was evaluated by analyzing the percentage of RSD of the internal standard substance in the six repeated tests. The IBM SPSS Statistics 21 software was used to analyze metabolites with one-way ANOVA followed by a Tukey’s test at a significance level of *p* < 0.05.

HCA were carried out with IBM SPSS Statistics 21 software based on average areas calculated from principal ions of all the compounds with varying content. In an agglomerative HCA, each sample begins as a separate cluster and the algorithm proceeds to combine them until all of the samples belong to one cluster. Two parameters need to be considered when performing hierarchical clustering. The first one is a similarity measurement, such as Euclidean distance, Pearson’s correlation, or Spearman’s rank correlation. The other parameter is a clustering algorithm, such as average linkage (clustering using the central observations), complete linkage (clustering using the farthest pair of observations between the two groups), single linkage (clustering using the closest pair of observations), or Ward’s linkage (clustering to minimize the sum of squares of any two clusters). Clustering results are shown as dendrograms. Here, Euclidean distance and Ward’s linkage were used.

## Additional files

Additional file 1: The quantitative data for the HCA. (XLSX 61 kb)

Additional file 2: The qualitative informations of commonly-induced metabolites in Table 2. (XLSX 29 kb)

## Data Availability

All data and materials are available on request for academic use.

## Abbreviations

GC–MS: gas chromatography–mass spectrometry
HCA: hierarchical clustering analysis
Ip: ironsulphur protein
MIC: minimum inhibitory concentration
MOA: mode(s) of action
PCA: principal components analysis
QoI: quinone outside Inhibitors
RSD: relative standard deviation
SDHIs: succinate dehydrogenase inhibitors
TMCS: trimethylchlorosilane
